# Combating HIV stigma in health care settings: what works?

**DOI:** 10.1186/1758-2652-12-15

**Published:** 2009-08-06

**Authors:** Laura Nyblade, Anne Stangl, Ellen Weiss, Kim Ashburn

**Affiliations:** 1 International Center for Research on Women, Washington, DC, USA

## Abstract

The purpose of this review paper is to provide information and guidance to those in the health care setting about why it is important to combat HIV-related stigma and how to successfully address its causes and consequences within health facilities. Research shows that stigma and discrimination in the health care setting and elsewhere contributes to keeping people, including health workers, from accessing HIV prevention, care and treatment services and adopting key preventive behaviours.

Studies from different parts of the world reveal that there are three main immediately actionable causes of HIV-related stigma in health facilities: lack of awareness among health workers of what stigma looks like and why it is damaging; fear of casual contact stemming from incomplete knowledge about HIV transmission; and the association of HIV with improper or immoral behaviour.

To combat stigma in health facilities, interventions must focus on the individual, environmental and policy levels. The paper argues that reducing stigma by working at all three levels is feasible and will likely result in long-lasting benefits for both health workers and HIV-positive patients. The existence of tested stigma-reduction tools and approaches has moved the field forward. What is needed now is the political will and resources to support and scale up stigma-reduction activities throughout health care settings globally.

## Review

A renewed global focus on HIV prevention, combined with a massive roll out of antiretroviral therapy, has focused worldwide attention on the ability of health facilities to deliver critical prevention, care and treatment services to a growing client population. HIV-related stigma and discrimination are now recognized as key barriers both to the delivery of quality services by health providers and to their utilization by community members and health providers themselves.

Unfortunately, the health sector is one of the main settings where HIV-positive individuals and those perceived to be infected experience stigma and discrimination [1,2]. Studies show that HIV-related stigma in this context is pernicious, and that its physical and mental health consequences to patients can be damaging [3-7]. Reducing HIV-related stigma in health settings should be a leading priority for health care managers. Yet little attention has been paid to this issue, particularly in low-resource countries grappling with burgeoning HIV epidemics.

Three main challenges contribute to this lack of attention. First, there is limited recognition of the important link between HIV-related stigma and public health outcomes, such as patient quality of care, and health workforce capacity. Stigma and discrimination by health workers compromises their provision of quality care, which is critical for helping patients adhere to medications and maintain their overall health and wellbeing. Stigma also acts as a barrier to accessing services both for the general population, as well as health providers themselves. This can have serious implications for health workers and health facilities when HIV-infected health workers delay or avoid care and become seriously ill or die, causing further strain on an overburdened health care system. Second, there is insufficient capacity among health care managers regarding how to effectively address stigma and discrimination through programmes and policies. Third, there is a persistent misconception that stigma is too pervasive a social problem to effectively change [8].

The purpose of this paper is to provide information and guidance to those in the health care setting, not only about why it is important to combat HIV-related stigma, but also how to successfully address its causes and consequences within health facilities. The paper begins by defining stigma and discussing how stigma manifests in the health care setting and its effects on patients, staff and the health care facility. It also highlights how stigma affects health workers living with HIV.

The paper then presents evidence-based fundamentals that should be applied when designing stigma-reduction efforts. This is followed by a discussion of specific strategies that have been particularly effective at reducing stigma in health facilities and addressing the needs of HIV-positive health workers, as well as tools and resources that are available for developing and implementing stigma reduction efforts in health care settings.

### Defining stigma

UNAIDS defines HIV-related stigma and discrimination as: "... a 'process of devaluation' of people either living with or associated with HIV and AIDS ... Discrimination follows stigma and is the unfair and unjust treatment of an individual based on his or her real or perceived HIV status."[9]

Stigma often heightens existing prejudices and inequalities. HIV-related stigma tends to be most debilitating for people who are already socially marginalized and closely associated with HIV and AIDS, such as sex workers, men who have sex with men, injecting drug users, and prisoners [10,11].

Men and women may experience different forms and intensities of stigma. For example, among HIV-positive South African adults surveyed, men reported greater self-abasing beliefs and adverse social reactions to their HIV status than women [12]. Conversely, other studies have shown that women are particularly vulnerable to stigma, including violence, one of the harshest and most damaging forms of stigma [13-18].

## Stigma in health facilities

### Manifestations and ramifications

There are many ways in which HIV-related stigma manifests in health care settings. A study in Tanzania documented a wide range of discriminatory and stigmatizing practices, and categorized them broadly into neglect, differential treatment, denial of care, testing and disclosing HIV status without consent, and verbal abuse/gossip [19]. Similarly, a study in Ethiopia found that common forms of stigma in health facilities were designating patients as HIV positive on charts or in wards, gossiping about patients' status, verbally harassing patients, avoiding and isolating HIV-positive patients, and referring patients for HIV testing without counselling [17].

In Indian hospitals, stigma and discrimination manifested as health workers informing family members of a patient's HIV status without his or her consent, and doing the following only with HIV-positive patients: burning their bedding upon discharge, charging them for the cost of infection control supplies, and using gloves during all interactions, regardless of whether physical contact occurred [20].

Stigma and discrimination in the health care setting and elsewhere contribute to keeping people, including health providers, from adopting HIV preventive behaviours and accessing needed care and treatment. Fear of being identified as someone infected with HIV increases the likelihood that people will avoid testing for HIV, disclosing their HIV status to health care providers and family members, or seeking treatment and care, thus compromising their health and wellbeing.

With its potentially devastating consequences on care-seeking behavior, stigma represents a major "cost" for both individuals and public health. Both experienced and perceived stigma and discrimination are associated with reduced utilization of prevention services, including programmes to prevent mother to child transmission [21-25], HIV testing and counselling [26-30], and accessing care and treatment [31].

In addition, research has demonstrated that the experience or fear of stigma often results in postponing or rejecting care, seeking care far from home to protect confidentiality, and nonadherence to medication. For example, studies in Senegal and Indonesia documented that men who have sex with men and injecting drug users, respectively, often avoid or delay accessing HIV-related services, including treatment for other sexually transmittted diseases, for fear of public exposure and discrimination by health workers [28,32].

Likewise, reseachers in Botswana and Jamaica found that stigma leads many people to seek testing and treatment services late in the progression of their disease, often beyond the stage of optimal drug intervention [30,33]. To conceal use of antriretroviral medications, HIV-positive individuals in South Africa reported grinding drugs into powder and not taking medication in front of others, which can result in inconsistent dosing [34].

As mentioned, health care providers themselves may be reluctant to access the same testing, care and treatment they provide to their patients due to fear of stigma in the workplace and in the communities they serve [35]. A study in South Africa and Botswana found that health workers struggle with self-stigma regarding a potential HIV diagnosis, as well as fear of stigmatizing attitudes and behaviours from their colleagues, which contribute to a lack of uptake of HIV testing and early treatment, if needed [36].

In Zambia, health workers report knowing peers who are hiding their HIV status, are afraid to talk about their situation to others, and are suffering in silence [37]. One indication of health workers' fears around HIV testing is their interest in self testing. A national study of health providers in Kenya found that nearly three-quarters would be interested in testing themselves for HIV, if such an option existed. Interest was greatest among those who had never tested, among medical doctors, and among health providers from the province with the highest prevalence of HIV in the country. The main reasons given for their interest are that self testing eliminates a potential breach in confidentiality, and pre-empts stigma and suspicion from colleagues since they would not know that someone had tested for HIV [38].

While health workers living with HIV may face the same kinds of stigma as their patients because of perceived improper or immoral behaviours, their self-blame and shame may be compounded by their relatively higher social and educational status in the community. As noted by one hospital manager in a Zambia study, "In the end it was us that were stigmatizing ourselves. I feel people that are more educated, like nurses, find it most difficult to discuss and disclose their status ..." [37].

Health providers interviewed in another study in Zambia report that medical personnel who become infected with HIV are commonly seen as failures in the community [39]. Nurses in Thailand expressed concern that their professional status would not give them the benefit of the doubt from their colleagues regarding whether they acquired their infection occupationally or through sex or drugs. For them, women with HIV violate gender norms and thus are guilty of being promiscuous [40]. This suggests that health providers fear a loss of status and moral integrity if their peers find out they are HIV positive.

### Immediately actionable causes of HIV-related stigma

Research conducted among general populations around the world has revealed three immediately actionable key causes of HIV-related stigma in the community setting: lack of awareness of what stigma looks like and why it is damaging; fear of casual contact stemming from incomplete knowledge about HIV transmission; and values linking people with HIV to improper or immoral behaviour [2,41-43].

Similarly among health care workers, research suggests that fear of casual contact and moral judgements contributes to stigma and discrimination directed at clients living with HIV. Studies in Nigeria, Mexico, Ethiopia and Tanzania [2,14,44-48] have found high levels of fear of contagion among health workers, which is related to a lack of understanding of how HIV is and is not transmitted, and how to protect oneself in the workplace through universal precautions.

In India, a study of hospital workers found that those who expressed greater agreement with stigmatizing statements about people living with HIV and hospital discriminatory practices were more likely to have incorrect knowledge about HIV transmission [20]. With regard to moral judgements, studies have demonstrated that the assumption that people with HIV have conducted themselves in some improper or immoral way contributes to health workers' negative attitudes toward HIV-positive people and permeates client-provider interactions. In Nigeria, results of a study among nurses and laboratory technicians showed that 35% felt that HIV-positive people deserved being infected as punishment for their "sexual misbehaviours" [45]. Similarly in Mexico, three-quarters of health providers surveyed thought people with HIV bore responsibility for having HIV [48].

### The value of a supportive, stigma-free environment

There is increasing evidence of the value of supportive and de-stigmatizing HIV services in different HIV prevalence and socio-cultural settings.

In China, health care workers who provide medical and emotional support are viewed favourably by HIV-positive patients and as critical to their ability to stay healthy, especially in the light of family isolation due to intense HIV stigma [49]. Cataldo (2008) describes new forms of citizenship and socio-political inclusion among low-income people living with HIV in Brazil, a country lauded for its policy of free universal access to antiretroviral therapy [50]. He documents close and supportive relationships between health practitioners and their clients, and between the health system and community non-governmental organizations that offer meetings, workshops, legal advice and support groups. Through de-stigmatizing care and treatment services they receive from the health system and related services in the community, clients are encouraged to claim further rights to be involved in decision-making processes, to achieve greater social inclusion, and to challenge stigma in the workplace and within families.

## Reducing stigma in health facilities

### A focus on the individual, environmental and policy levels

Although stigma is a pervasive and daunting problem in the health care setting, much can be done to address its causes and consequences. A key lesson that has emerged from recent research and field experiences is that to combat stigma in the health care setting, interventions must focus on the individual, environmental and policy levels [3,51].

#### Individual level

At the individual level, increasing awareness among health workers of what stigma is and the benefits of reducing it is critical. Raising awareness about stigma and allowing for critical reflection on the negative consequences of stigma for patients, such as reduced quality of care and patients' unwillingness to disclose their HIV status and adhere to treatment regimens, are important first steps in any stigma-reduction programme. A better understanding of what stigma is, how it manifests and what the negative consequences are can help reduce stigma and discrimination and improve patient-provider interactions.

Health workers' fears and misconceptions about HIV transmission must also be addressed. Fear of acquiring HIV through everyday contact leads people to take unnecessary, often stigmatising actions. Thus programmes need to provide health workers with complete information about how HIV is and is not transmitted and how practicing universal precautions can allay their fears. In addition to basic HIV epidemiology, health workers must be able to understand the occupational risk of HIV infection relative to other infectious diseases that are more highly transmissable and commonly found in heath care settings.

Understanding the association of HIV and AIDS with assumed immoral and improper behaviours is essential to confronting perceptions that promote stigmatizing attitudes toward individuals living with HIV. Programmes need to address the shame and blame directed at people with HIV by providing health providers with a safe space to reflect on the underlying values that lead to the shame and blame. It is important for health care workers to disassociate persons living with HIV from the behaviours considered improper or immoral that are often associated with HIV infection.

#### Environmental level

In the physical environment, programmes need to ensure that health workers have the information, supplies and equipment necessary to practice universal precautions and prevent occupational transmission of HIV. This includes gloves for invasive procedures, sharps containers, adequate water and soap or disinfectant for handwashing, and post-exposure prophylaxis in case of work-related, potential exposure to HIV. Posting relevant policies, handwashing procedures or other critical information in key areas in the health care setting enables health workers to maintain better quality of patient care.

#### Policy level

The lack of specific policies or clear guidance related to the care of patients with HIV reinforces discriminatory behaviour among health workers. Health facilities need to enact policies that protect the safety and health of patients, as well as health workers, to prevent discrimination against people living with HIV. Such policies are most successful when developed in a participatory manner, clearly communicated to staff, and routinely monitored after implementation.

Several studies have shown that stigma reduction activities in hospitals, based on the principles we have outlined, have led to positive changes in health providers' knowledge, attitudes and behaviours, and better quality of care for HIV-positive patients [3,51,52].

For example, following a stigma-reduction intervention in four Vietnamese hospitals [51], the mean score on both a fear-based and a value-based stigma index decreased significantly among hospital workers (p < 0.05). Additionally, there was a significant reduction in reporting of discriminatory behaviours and practices by hospital workers. For example, the percentage of hospital workers reporting the existence of labels indicating HIV status on files declined from 56% to 31% (p < 0.001) in one hospital, and from 31% to 17% in another (p < 0.002). During monitoring visits, various positive changes were observed (e.g., improvements in the use of universal precautions, increased voluntary HIV testing of patients and informing patients of their HIV status, and a reduction in the marking of files and beds with the patient's HIV status).

The intervention accomplished this reduction in stigma and discrimination within six months through the following programmatic steps:

1) Implementation of a brief survey to document the need for action to reduce stigma and guide the design of the intervention

2) Establishment of a steering committee to plan the intervention

3) A flexibly scheduled 2 1/2 day participatory training for all hospital staff (from cleaners to clerks to doctors), which focused on increasing knowledge and awareness of HIV, universal precautions, and fear-based and value-based stigma, including what stigma looks like in the health care setting

4) Participatory drafting and negotiation by all staff of a hospital policy to foster staff safety and a stigma-free atmosphere

5) Provision of materials and supplies to facilitate the practice of universal precautions.

This and other intervention studies in hospitals [3,52] suggest a number of promising pathways and approaches for tackling the problem at the individual, environmental and policy levels. Stigma reduction fundamentals for the hospital setting, outlined below, are also applicable in other health care settings, such as primary care clinics and health posts.

### Involve all staff members, not just health professionals, in training and in crafting policy

Reaching everyone with whom a patient comes in contact (e.g., doctors, nurses, guards, cleaners and administrative staff) helps ensure ownership of the stigma-reduction process and a unified response by the health care facility.

### Use participatory methods

Participatory methods such as games, role plays, exercises and group discussions create a non-judgemental environment that allows participants to explore personal values and behaviours, while improving their knowledge and awareness. It also creates a sense of ownership in the process of developing stigma-reduction strategies in the health care setting.

A variety of tested tools exist from which to find participatory exercises on stigma reduction to build your programme. They include: Understanding and Challenging HIV Stigma: A Toolkit for Action [53], a general tool that has worked well in health facilities, as well as two participatory tools focused specifically on the health care setting: Safe and Friendly Health Facility Trainers Guide [54], and Reducing HIV Stigma and Gender-Based Violence: Toolkit for Health Care Providers in India [55].

### Provide training on both stigma and universal precautions

Equipping health workers with the knowledge and skills necessary to protect themselves from occupational transmission of HIV is a key step in addressing fear-based stigma. But health workers also must be provided with the supplies necessary (e.g., gloves, gowns, water and disinfectant solution) so that they can take appropriate steps to ensure staff and patient safety.

### Involve individuals living with HIV

Showing that HIV has a "human face" helps health workers to better understand stigma and its insidious impact on individuals and families. Involving members of socially marginalized groups who are HIV positive, such as men who have sex with men, sex workers, and injecting drug users, also helps to address the additional social stigmas they face on top of HIV-related stigma.

When designing a training programme, it is important to tap into existing networks of people living with HIV to identify individuals to take part in training activities, as well as to provide adequate preparation and training to these individuals to equip them for the role they will play in training (e.g., testimonials and co-facilitation). An important group to have represented, if possible, is health care workers living with HIV.

### Periodically monitor stigma among health workers

One way to ensure that this happens is by enacting health care setting regulations that mandate the monitoring of health worker attitudes and behaviours to assess progress. It is also important to establish anti-stigma policies and benchmarks that health facilities can use for assessing their efforts. For example, the government of Vietnam is currently updating its national hospital regulations to include stigma reduction, and is developing a tool that hospitals can use to determine the extent to which they are in compliance.

### Take advantage of existing tools

We have described two participatory resources that have been tested and shown to be effective in different contexts for training health workers, as well as one for other groups. With regard to programme planning and monitoring, a hospital-based intervention in India produced a tool that health workers can use to assess the extent to which a facility complies with anti-stigma and discrimination standards. This is the PLHA-friendly checklist [56], which can be used to catalyze action in a given facility and also as an evaluation tool. Another tool for training health care workers is: Reducing Stigma and Discrimination Related to HIV and AIDS: Training for Health Care Workers [57].

### Address the needs of HIV-infected health workers

Health facilities should respond in a multi-faceted way to address HIV-positive health workers' fear of stigma and loss of confidentiality. The response should include private and confidential counselling and testing services, access to antiretroviral therapy, and professional and emotional support, either on the premises or at a convenient location. Also important are the enactment and enforcement of anti-discrimination policies to protect health workers living with HIV [36].

## The way forward: investing in stigma reduction

This paper highlights the importance of combating stigma in health facilities and discusses several feasible activities that have been shown to reduce stigma by health providers. Stigma reduction in health facilities, as we have argued, has important implications for improving patient-provider interactions, improving quality of care, and creating a safe and supportive space for clients that can help them deal with, and in some cases, challenge stigma from family and community members.

Stigma reduction is also a first step in creating services to address the needs of HIV-positive health workers. The availability of tested stigma-reduction tools and approaches has moved the field forward. What is needed now is the political will and resources to support and scale up stigma reduction activities throughout health care settings globally. Given the detrimental effect of stigma on both individual health and wellbeing and public health outcomes, it is clear that health care managers cannot afford inaction any longer.

## Competing interests

The authors declare that they have no competing interests.

## Authors' contributions

LN and AS conceived the manuscript. EW drafted the manuscript based on papers, technical reports and presentations by LN, AS, and KA, who reviewed the draft and gave comments. All authors read and approved the final manuscript.
